# Spatiotemporal distribution of climate-sensitive disease incidences in ethiopia: a longitudinal retrospective analysis of Malaria, Meningitis, Cholera, Dysentery, Leishmaniasis and Dengue fever between 2010 and 2022/2023

**DOI:** 10.1186/s12889-024-18054-3

**Published:** 2024-03-04

**Authors:** Gizeaddis Lamesgin Simegn, Mizanu Zelalem Degu, Worku Birhanie Gebeyehu, Asaye Birhanu Senay, Janarthanan Krishnamoorthy, Geletaw Sahle Tegenaw

**Affiliations:** 1 Biomedical Imaging Unit, School of Biomedical Engineering, Jimma Institute of Technology, Jimma University, Jimma, Ethiopia; 2 Artificial Intelligence & Biomedical Imaging Research Lab, Jimma Institute of Technology, Jimma University, Jimma, Ethiopia; 3 Faculty of Computing, Jimma Institute of Technology, Jimma University, Jimma, Ethiopia; 4 Department of Health Policy and management, Jimma Institute of Health, Jimma University, Jimma, Ethiopia

**Keywords:** Cholera, Climate-sensitive, Dengue fever, Disease incidence, Dysentery, Epidemiology, Ethiopia, Leishmaniasis, Malaria, Meningitis, Prevalence patterns, Public health, Spatiotemporal distribution

## Abstract

**Background:**

Understanding the temporal and geographic distribution of disease incidences is crucial for effective public health planning and intervention strategies. This study presents a comprehensive analysis of the spatiotemporal distribution of disease incidences in Ethiopia, focusing on six major diseases: Malaria, Meningitis, Cholera and Dysentery, over the period from 2010 to 2022, whereas Dengue Fever and Leishmaniasis from 2018 to 2023.

**Methods:**

Using data from Ethiopian public health institute: public health emergency management (PHEM), and Ministry of Health, we examined the occurrence and spread of each disease across different regions of Ethiopia. Spatial mapping and time series analysis were employed to identify hotspots, trends, and seasonal variations in disease incidence.

**Results:**

The findings reveal distinct patterns for each disease, with varying cases and temporal dynamics. Monthly wise, Malaria exhibits a cyclical pattern with a peak during the rainy and humid season, while Dysentery, Meningitis and Cholera displays intermittent incidences. Dysentery cases show a consistent presence throughout the years, while Meningitis remains relatively low in frequency but poses a potential threat due to its severity. Dengue fever predominantly occurs in the eastern parts of Ethiopia. A significant surge in reported incident cases occurred during the years 2010 to 2013, primarily concentrated in the Amhara, Sidama, Oromia, Dire Dawa, and Benishangul-Gumuz regions.

**Conclusions:**

This study helps to a better understanding of disease epidemiology in Ethiopia and can serve as a foundation for evidence-based decision-making in disease prevention and control. By recognizing the patterns and seasonal changes associated with each disease, health authorities can implement proactive measures to mitigate the impact of outbreaks and safeguard public health in the region.

## Introduction

Infectious diseases remain a significant public health concern in Ethiopia, and their impact on the population’s health and well-being cannot be underestimated. Among the array of infectious diseases that continue to pose challenges, Malaria, Meningitis, Cholera, Dengue fever, Dysentery and Leishmaniasis stand out due to their potential for widespread outbreaks and severe health consequences [1–4]. These diseases are among the climate sensitive that are dominant in Sub-Saharan countries. This vulnerability is particularly evident due to the region’s diverse climate zones and socio-economic challenges. Diseases like malaria and dengue fever flourish as warmer temperatures accelerate mosquito breeding, while cholera outbreaks coincide with heavy rainfall and flooding. Vector-borne diseases, waterborne diseases, respiratory ailments worsened by air quality changes, and malnutrition-linked illnesses further highlight the intricate links between climate and health in this context [5–8]. Addressing these challenges requires robust healthcare systems, improved sanitation, vector control, disease surveillance, and climate adaptation strategies to safeguard public health. Monitoring the spatiotemporal distribution of these diseases is vital for understanding their epidemiology, identifying high-risk regions, and formulating targeted interventions to effectively control and prevent disease outbreaks.

Over the past decade, Ethiopia has made commendable efforts to improve its healthcare infrastructure and strengthen disease surveillance systems. As a result, there has been notable progress in reducing the incidence of certain infectious diseases, such as Malaria [9]. Despite these achievements, the country still faces significant challenges in combating other infectious diseases, including Meningitis [10, 11] and Dysentery [12, 13]. Additionally, the frequent occurrence of Dengue Fever in specific areas, especially in easter part of Ethiopia has raised concerns about the potential for outbreaks [14].

Spatial and temporal analyses of disease outbreaks have emerged as essential tools in public health research, allowing for the identification of disease hotspots and trends that may not be evident through traditional epidemiological approaches [15–18]. These analytical techniques provide valuable insights into the underlying factors contributing to disease transmission and can significantly influence evidence-based policies and interventions.

Over the years, several research studies have focused on investigating the spatial distribution and epidemiology of specific infectious diseases in Ethiopia. For example, Alene et al. [19] utilized Bayesian model-based geostatistical framework for a survey of HIV, TB and malaria, harnessing the power of high-resolution spatial covariates to predict continuous prevalence surfaces specific to each disease, along with their co-distribution. The findings revealed significant association of the spatial distribution of the diseases with healthcare access, demographic and climatic factors. Another study by Warkaw et al. [20]. explored the spatial patterns and predictors of malaria distribution in Ethiopia. Their research utilized Global Moran’s I and Moran scatter plots and local Moran’s I statistic to determine the distribution and investigate the predictors of malaria. The study identified that the occurrence of malaria in Ethiopia exhibited a spatial arrangement linked to socio-economic, demographic, and geographic risk factors. Spatial clustering of malaria cases was evident across all regions, with variations in the risk of clustering observed among these regions. Likewise, in a study by Beyene et al. [21], a comprehensive analysis was undertaken to investigate the spatial, temporal, and spatiotemporal dynamics of under-five diarrhea in Southern Ethiopia. The study involved the calculation of annual diarrhea incidence rates at the district level, encompassing the utilization of incidence rate calculations and seasonal trend analysis. Utilizing data retrospectively obtained from the Health Management Information System (HMIS), covering under-five diarrheal morbidity reports from July 2011 to June 2017 in the Sidama Zone (current Sidama Region), the research discerned distinct patterns. The outcomes of this study underscored the non-random distribution of childhood diarrhea across space and time, revealing an overarching increasing trend marked by seasonal fluctuations.

These studies collectively demonstrate the importance of spatial and temporal analyses in understanding disease epidemiology in Ethiopia. By pinpointing high-risk areas and identifying contributing factors, these research endeavors have played a vital role in guiding public health policies and interventions for various infectious diseases in the country. However, there remains a gap in comprehensive research that simultaneously explores multiple diseases and their spatiotemporal patterns over an extended period.

The primary objective of this study is to conduct a comprehensive analysis of the spatiotemporal distribution of Malaria, Meningitis, Cholera, Dysentery, Leishmaniasis and Dengue fever Incidents between 2010 and 2023. By examining the temporal trends, seasonal variations, and geographic patterns of each disease, we aim to gain a deeper understanding of their dynamics and identify potential risk factors associated with disease transmission and incidences.

Through this research, we aspire to contribute valuable insights to the existing body of knowledge on disease epidemiology in Ethiopia. By shedding light on the spatiotemporal distribution of these infectious diseases, we hope to provide crucial information to public health policymakers and stakeholders. This, in turn, will aid in formulating targeted and efficient strategies for disease surveillance, prevention, and control, ultimately reducing the burden of infectious diseases on the Ethiopian population. In this paper, the term “disease incidence” encompasses any reported occurrence of a disease, whether it is a newly emerging one or one that has already occurred.

## Methods

### Ethical considerations

Approval for the research was granted by the ethical review board of the Jimma Institute of Technology, with the reference number RPD/JIT/172/15.

### Data source

The main source of the study was a secondary data source collected from the Ethiopian public health institute (EPHI): public health emergency management (PHEM), and Ministry of Health (MoH). The EPHI dataset contains four diseases, including Malaria, Meningitis, Dysentery, and cholera/acute watery diarrhea (AWD). The dataset spans disease incidences across 12 regions of Ethiopia, encompassing Oromia, Southern Nations, Nationalities, and Peoples’ Region (SNNPR: which is now divided into South region, southwest region and central Ethiopia region), Amhara, Somali, Addis Ababa, Tigray, Afar, Benishangul Gumuz, Gambella, Sidama, Dire Dawa, and Harari. The disease incidences dataset comprised attributes including region name, zone name, woreda name, year, epidemic week, month, total malaria confirmed and clinical, total malaria outpatient cases, total malaria inpatient cases, total malaria inpatient deaths, total malaria suspected fever examined, post malaria rapid diagnostic tests (RDTs) or microscopy plasmodium falciparum (PF) outpatient cases, post malaria rapid diagnostic tests or microscopy plasmodium vivax (PV) outpatient cases, meningitis total cases, meningitis outpatient cases, meningitis inpatient cases, meningitis inpatient deaths, dysentery total cases, dysentery outpatient cases, dysentery inpatient cases, dysentery inpatient deaths’, cholera/AWD cases, cholera/AWD deaths. The Dengue Fever and Leishmaniasis data that span 2018 to 2023 was collected from MoH. The dataset spans disease incidences across three regions of Ethiopia, encompassing Amhara, Somali, and, Dire Dawa. The dataset comprises six attributes, including Religion, Disease, Zone, Year, Month, and Total Values.

### Study setting and design

Utilizing a longitudinal retrospective design, this study examines the prevalence and diffusion of a range of diseases including malaria, meningitis, cholera, dysentery, leishmaniasis, and dengue fever, across diverse Ethiopian regions, integrating longitudinal analysis with geospatial mapping and temporal trend assessment, this investigation synthesizes epidemiological data to reveal intricate patterns.

### Data preprocessing and analysis

The data pre-processing, visual inspection, and analysis of disease incidences datasets were conducted using Python. An analysis of missing values, outliers, and noisy data was conducted. The quality of the disease incidence data was examined using WHO data-quality review [22]. The disease incidence dataset was examined for completeness and timeliness, consistency, accuracy, and validity.

Further processing was performed on the EPHI disease incidence datasets columns, including total malaria confirmed and clinical, total malaria outpatient cases, total malaria inpatient cases, total malaria inpatient deaths, total malaria suspected fever examined, post malaria RDTs or microscopy PF outpatient cases, post malaria rapid diagnostic tests or microscopy PV outpatient cases, Meningitis total cases, outpatient cases, inpatient cases, inpatient deaths, dysentery total cases, dysentery outpatient cases, dysentery inpatient cases, dysentery inpatient deaths’, cholera/AWD cases, cholera/AWD deaths were further processed. Moreover, disease incidences with zero values were eliminated from the dataset. Whereas, seventy-five attributes were observed in the initial MoH Dengue Fever and Leishmaniasis disease incidence dataset, and six attributes remain after preprocessing.

The final disease incidence dataset that was used for analysis included the names of the region, zone name, woreda name, year, epidemic week, month, epidemic week, disease, category, type, and total value. Then, the occurrence and spread of each disease across different regions of Ethiopia was examined. Furthermore, spatial mapping and time series analysis were employed to identify hotspots, trends, and seasonal variations in disease incidence. The datasets from the two sources, such as EPHI and MoH, were integrated after the data preprocessing based on the religion, Disease, Zone, Year, Month, and Total Values attributes.

## Results

### Summary of regional disease incidence distribution

**Fig. 1:**
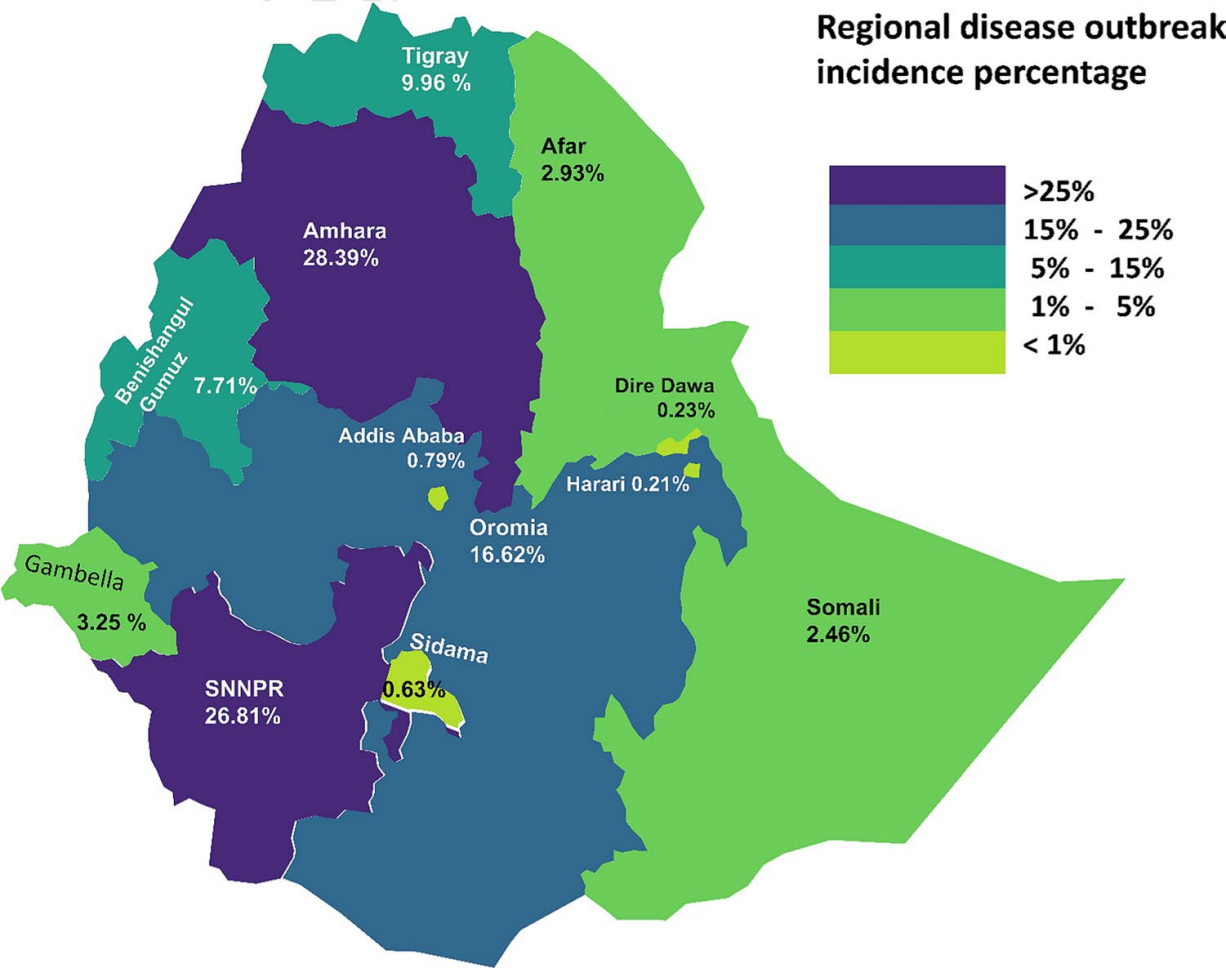
Summary of regional disease incidence distribution percentage in Ethiopian during the period of 2010 to 2022/2023

**Fig. 2:**
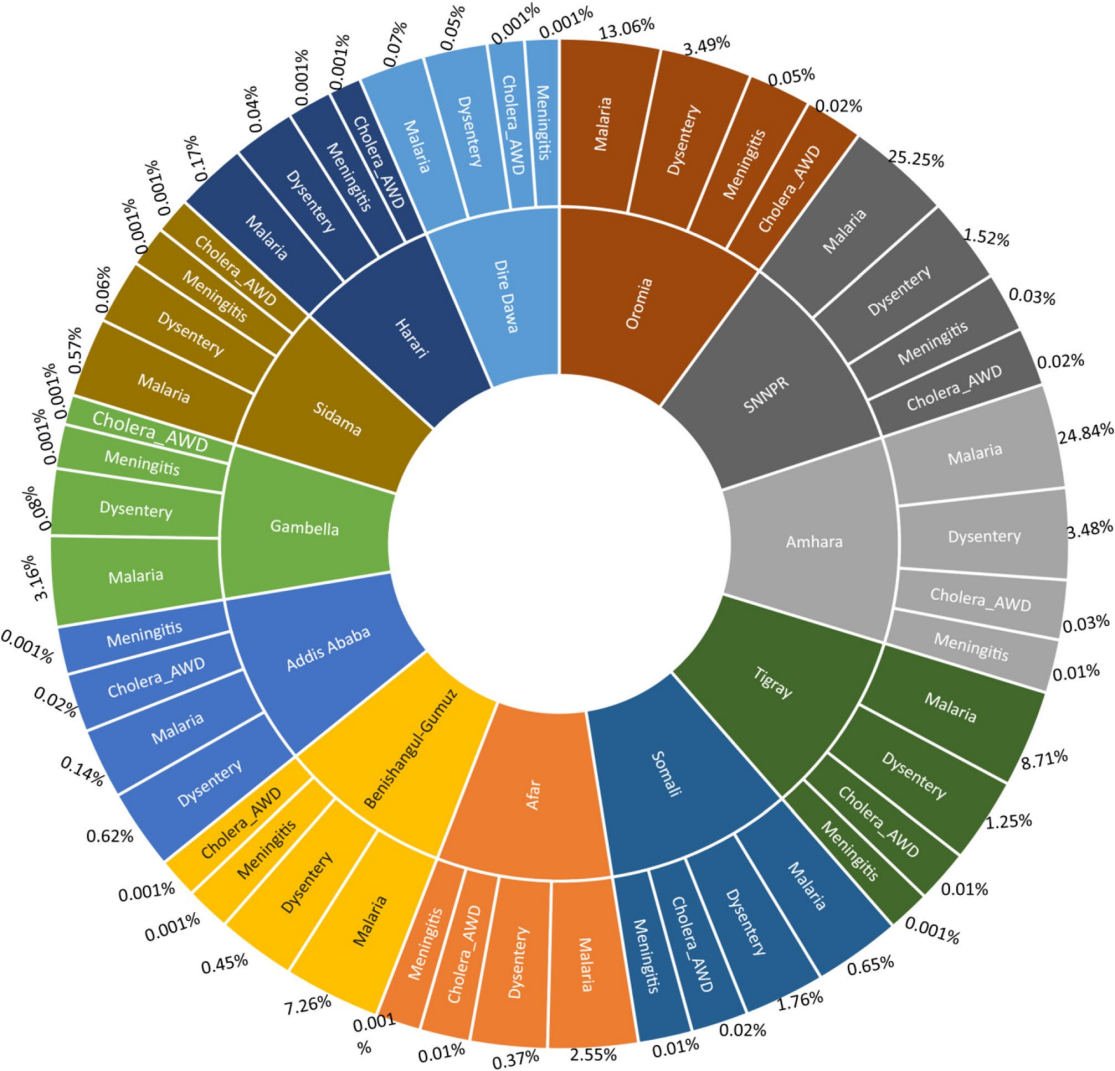
Detail regional disease incidence distributions (excluding Leishmaniasis and dengue fever) in Ethiopia during the period of 2010 to 2022/2023

Within the total dataset of disease incidence, the greatest incidence was recorded in the regions of Amhara, SNNPR, and Oromia, constituting percentages of 28.39, 26.81, and 16.62%, respectively. Detailed insights into the distribution of disease incidents in regions of Ethiopian can be found in Figs. 1 and 2.

### Comparison of total disease incidence distribution

**Fig. 3:**
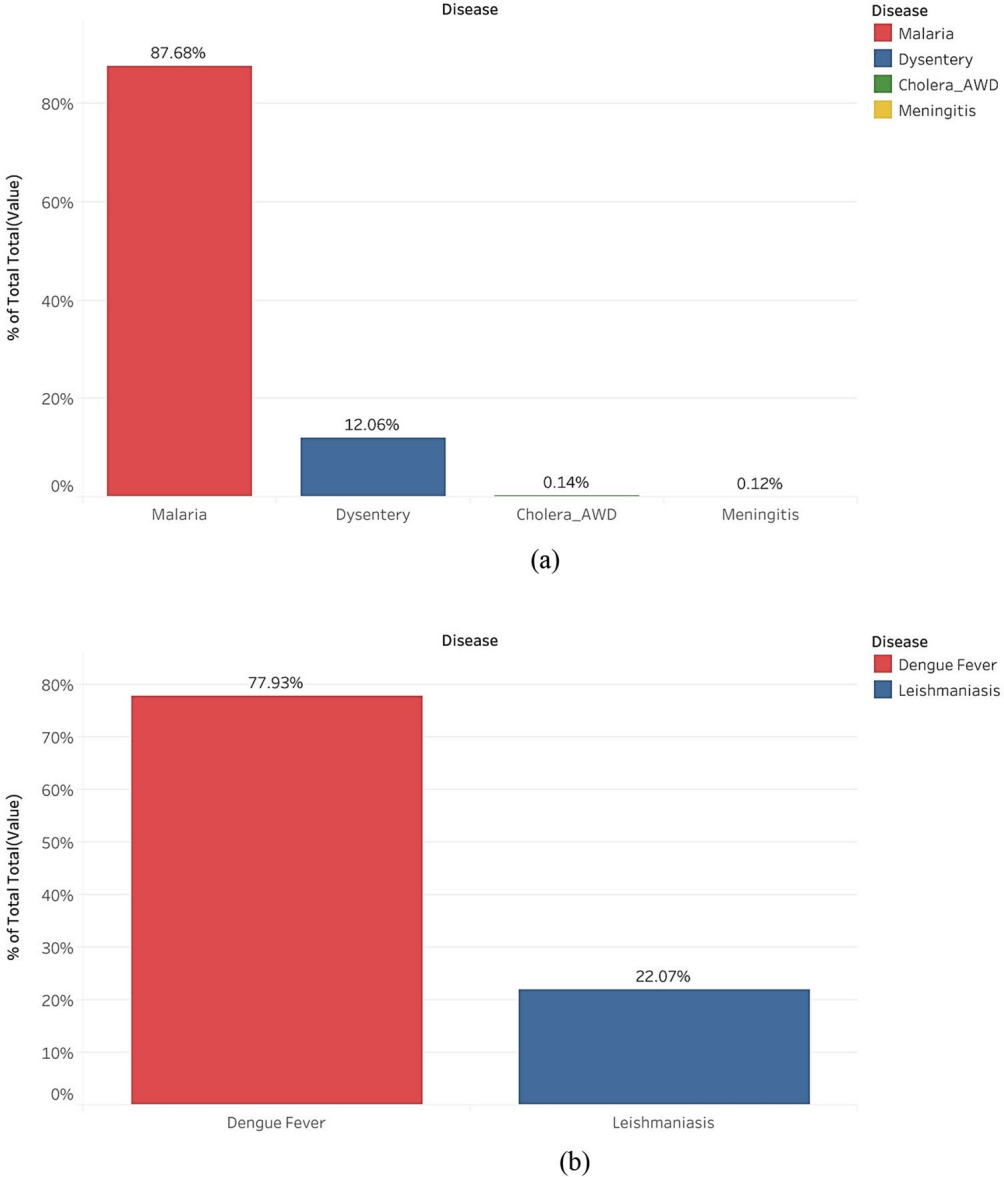
Comparision of total disease incidence rate in Ethiopia (**a**) Malaria, Dystenery, Cholera and Meningitis from 2010 to 2022, (**b**) Dengue fever and Leishmaniasis 2018 to 2023

In the data spanning from 2010 to 2022 (Fig. 3a), which includes information on malaria, dysentery, cholera, and meningitis, the highest observed incidences were related to malaria, accounting for 87.54%. Dysentery followed with a prevalence of 12.04%, while meningitis had the lowest reported incidence at just 0.12% of the total cases. In the case of leishmaniasis and dengue fever data (Fig. 3b) covering a five-year period from 2018 to 2023, dengue fever constituted the majority at 77.93%, whereas leishmaniasis accounted for approximately 22.07%.

### Disease incidence trend

#### Malaria disease incidence in Ethiopia from 2010 to 2022

In Addis Ababa and Tigray, the peak of disease incidence was observed in October, while in Afar, it occurred in December, as illustrated in Table 1. The highest incidence of malaria incidences was noted in November in the regions of Amhara, Benishangul-Gumuz, Dire Dawa, Oromia, Sidama, and SNNPR. Harari and Somali, on the other hand, experienced their highest malaria incidences in June.

**Fig. 4:**
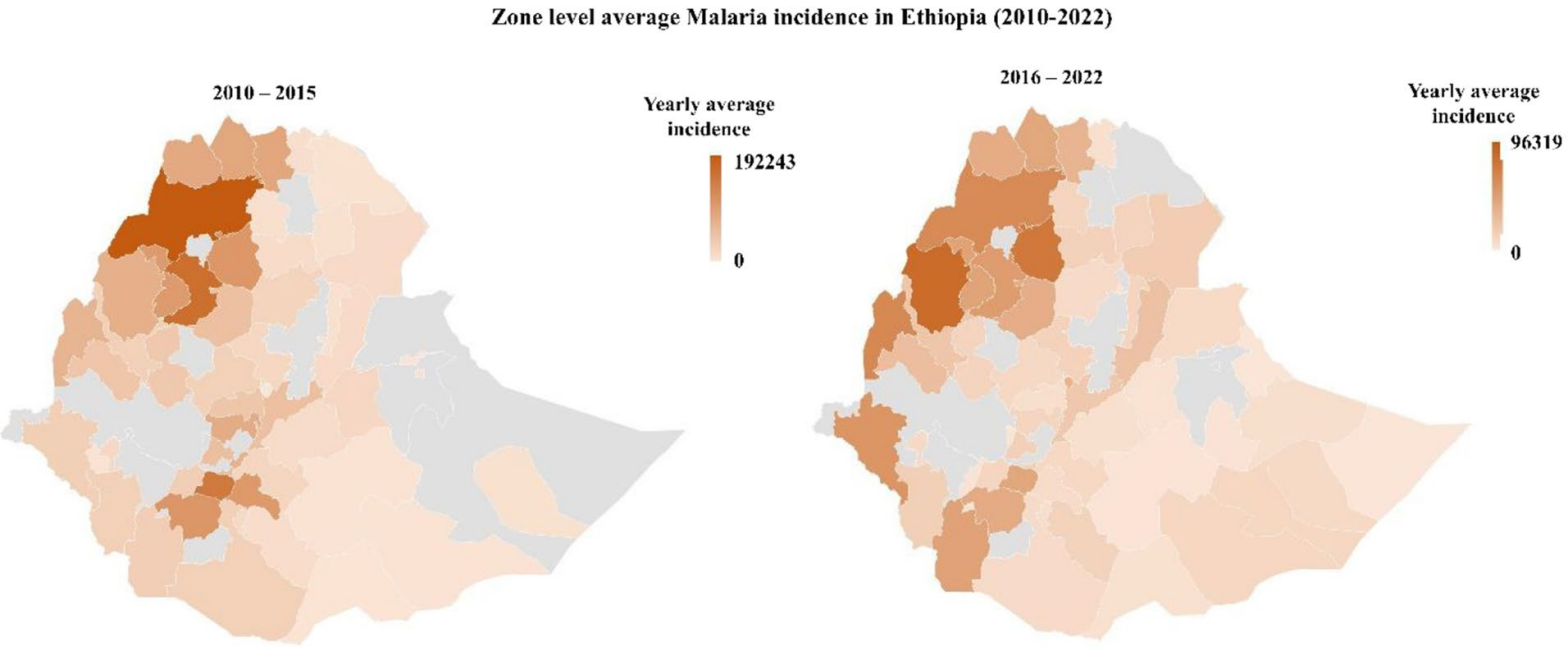
Zone level average Malaria incidence in Ethiopia between 2010 to 2022

Figure 4 shows the average incidence of malaria at the Zone level in Ethiopia from 2010 to 2022. In the latter period (2016–2022), there was a reduction in incidence, with the zonal average maximum decreasing from 192,243 cases to 96,319 cases.

**Table 1:** Monthly regional Malaria disease incidence in Ethiopia from 2010 to 2022

Region	Month												
	JAN	FEB	MAR	APR	MAY	JUN	JUL	AUG	SEP	OCT	NOV	DEC	
Addis Ababa	3035	2766	3466	2855	3392	3758	4095	3781	3563	**4780**	0	4157	
Afar	73,748	69,188	72,730	59,092	72,733	63,041	53,117	57,428	58,763	69,538	68,136	**77,378**	
Amhara	414,293	328,254	351,695	286,465	616,428	802,056	673,508	624,376	679,581	1,043,134	**1,118,656**	793,913	
Benishangul-Gumuz	147,987	106,672	117,003	93,025	134,022	229,132	219,089	183,528	169,817	292,880	**337,581**	229,140	
Dire Dawa	1182	1520	972	998	1556	1316	854	880	1308	4914	**4595**	2143	
Gambella	72,021	58,537	58,084	53,768	69,144	86,174	99,066	99,693	84,197	96,410	102,509	**104,744**	
Harari	2615	1865	1799	2733	4386	**6193**	6178	6125	5080	5853	5380	3973	
Oromia	266,304	211,190	239,300	223,435	315,834	338,717	355,675	364,256	353,744	482,798	**505,932**	410,177	
Sidama	4961	4386	5925	5469	8682	14,120	13,199	12,626	16,079	22,280	**37,031**	33,630	
SNNPR	558,025	518,026	606,513	640,332	733,501	689,718	697,714	670,938	603,022	737,760	**754,451**	649,665	
Somali	43,376	39,630	43,686	43,058	48,390	**52,787**	45,683	42,200	39,894	46,826	49,951	52,377	
Tigray	219,732	162,841	160,291	120,783	126,707	166,175	232,909	247,052	294,993	**375,156**	359,238	244,862	

The numbers highlighted in bold represent the month with the maximum disease incidence in each region

#### Dysentery disease incidence in Ethiopia from 2010 to 2022

As indicated in Table 2, the peak of dysentery incidence varied across regions: June in Addis Ababa, July in Amhara, August in Tigray and Afar, March in Benishangul-Gumuz, May in Oromia and Dire Dawa, April in Gambella, Harari, Sidama, and SNNPR, and December in Somalia.

**Fig. 5:**
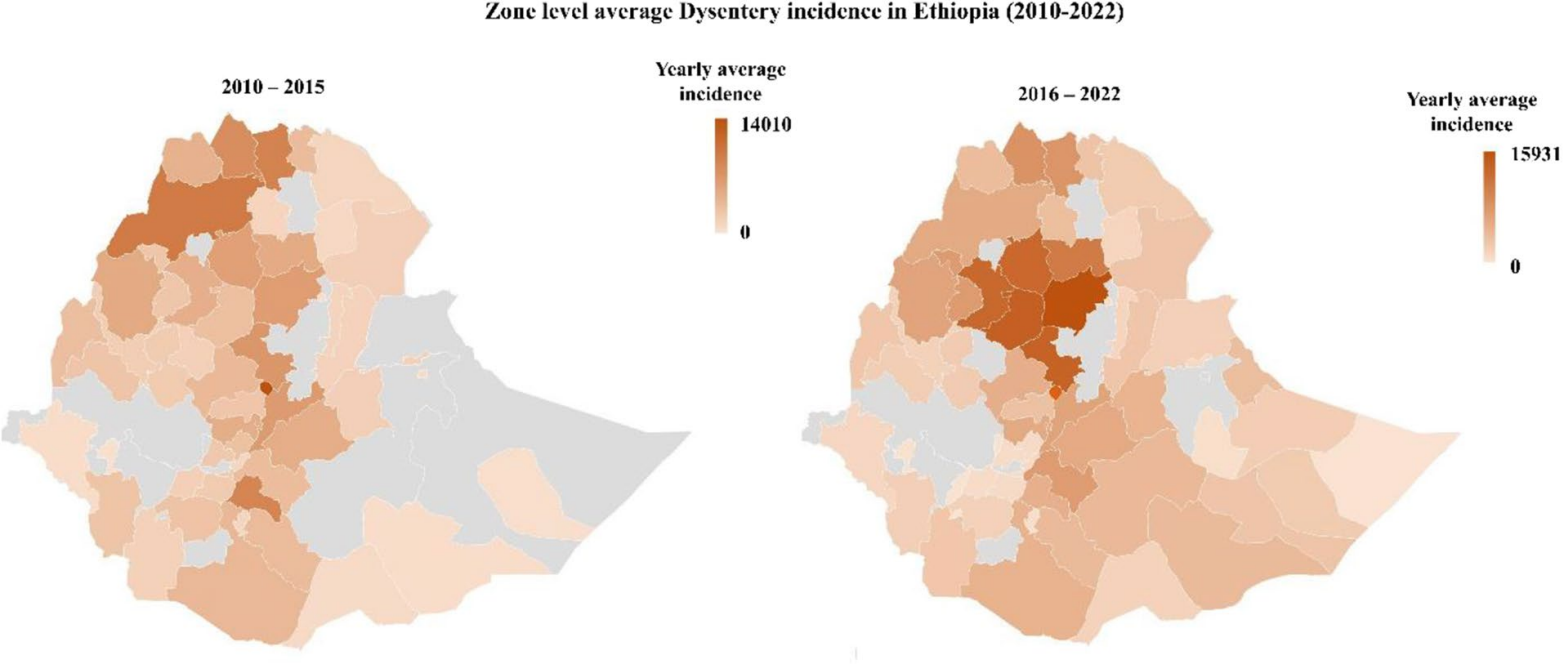
Zone level average Dysentery incidence in Ethiopia between 2010 to 2022

Figure 5 demonstrates the Zone level average Dysentery incidence in Ethiopia between 2010 and 2022. A slight increment has been observed in the second period (2016–2022) in zones of eastern Amhara and the Somali region.

**Table 2:** Monthly regional Dysentery disease incidence in Ethiopia from 2010 to 2022

Region	Month											
	JAN	FEB	MAR	APR	MAY	JUN	JUL	AUG	SEP	OCT	NOV	DEC
Addis Ababa	12,695	11,780	16,823	17,129	20,593	**20,655**	18,336	16,307	14,987	15,118	13,773	13,458
Afar	9100	8242	8863	8384	10,595	9183	9654	**11,154**	10,612	10,657	8261	9475
Amhara	55,764	57,461	75,750	79,156	104,206	111,639	**116,385**	124,091	101,306	103,120	83,106	72,462
Benishangul-Gumuz	10,348	10,346	**12,791**	9851	11,417	12,195	12,615	12,712	10,129	12,921	12,443	10,778
Dire Dawa	1196	1116	985	1169	**1987**	1659	1292	1438	1374	1407	1010	1031
Gambella	2149	2167	2371	**2509**	2376	2350	2074	1773	1729	1819	2036	2277
Harari	831	876	1316	**1561**	1502	1520	954	930	848	1003	966	814
Oromia	77,159	74,866	98,387	96,370	**108,679**	99,222	97,359	95,760	81,241	87,284	82,043	86,921
Sidama	962	1114	1611	**1984**	1581	1225	1316	1641	1650	1733	1434	1699
SNNPR	31,034	34,961	51,925	**55,018**	51,739	43,472	36,379	34,594	30,740	36,873	32,242	32,762
Somali	14,943	14,828	17,592	18,146	20,655	18,051	16,015	16,486	14,576	16,064	16,971	**18,938**
Tigray	25,425	23,061	26,975	25,544	30,956	36,612	41,556	**48,109**	42,530	35,884	28,254	22,752

The numbers highlighted in bold represent the month with the maximum disease incidence in each region

#### Cholera AWD disease incidence in Ethiopia from 2010 to 2022

As per the data presented in Table 3, the highest instances of Cholera Acute Watery Diarrhea (AWD) occurred in August in Addis Ababa, Dire Dawa, Harari, and Tigray. In Afar and Oromia, the peak was observed in June, while for Amhara and Sidama, it occurred in September. SNNPR, Gambella, Benishangul-Gumuz, and Somali reported their highest cases in January, November, October, and March, respectively.

**Fig. 6:**
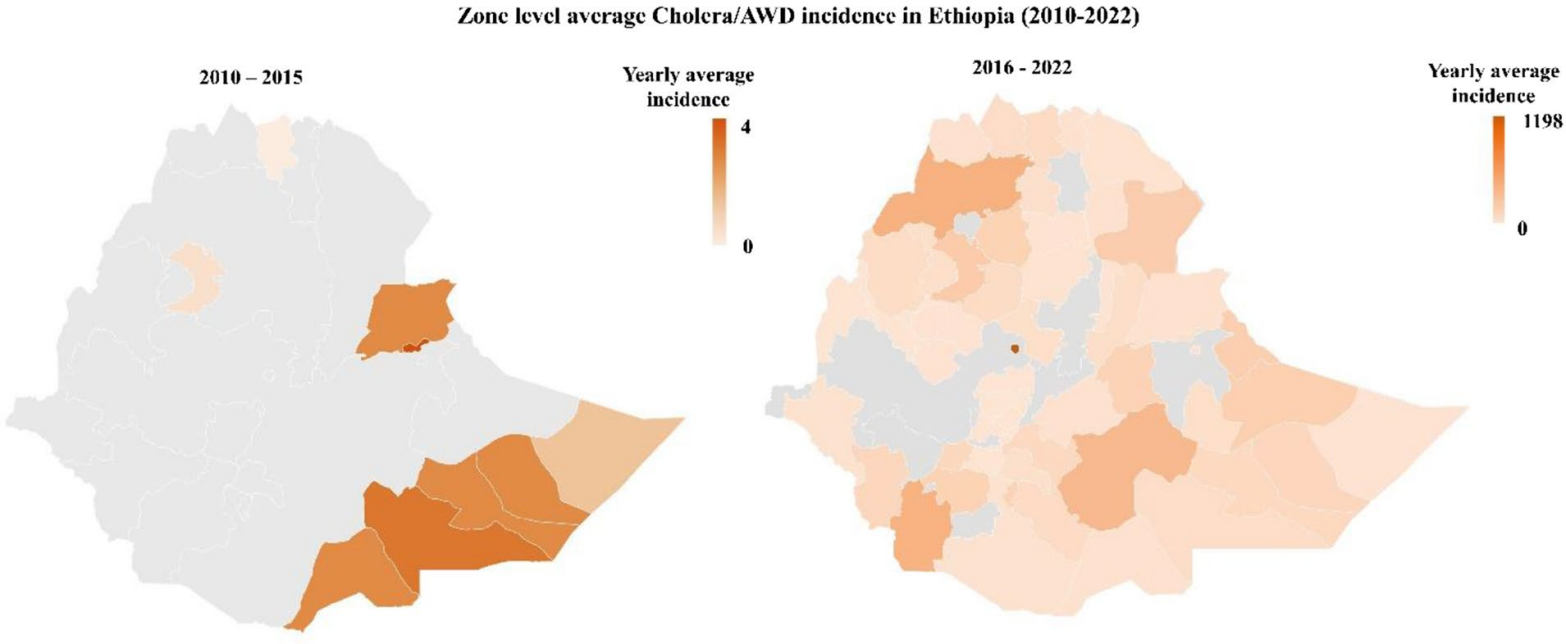
Zone level average Cholera/AWD incidence in Ethiopia between 2010 to 2022

Figure 6 depicts the average incidence of Cholera/AWD at the Zone level in Ethiopia from 2010 to 2022. During the latter period (2016–2022), the disease spread to most regions of Ethiopia.

**Table 3:** Monthly regional Cholera/AWD disease incidence in Ethiopia from 2010 to 2022

Region	Month												
	JAN	FEB	MAR	APR	MAY	JUN	JUL	AUG	SEP	OCT	NOV	DEC	
Addis Ababa	0	1		1	2	1634	3261	**2023**	576	171	5	1	
Afar	65	70	1	18	224	**372**	284	343	166	159	249	28	
Amhara	2	42	501	190	243	409	783	3115	**1523**	1364	472	110	
Benishangul-Gumuz	0	0	4	0	0	2	0	4	33	**298**	123	1	
Dire Dawa	15	1	0	0	0	11	0	**100**	34	81	0	30	
Gambella	0	0	0	0	0	0	0	0	0	2	**67**	25	
Harari	0	0	0	0	1	0	0	**110**	29	4	1	0	
Oromia	296	431	402	328	300	**1128**	685	372	985	469	662	910	
Sidama	0	0	0	0	0	0	0	61	**88**	41	33	21	
SNNPR	**1627**	376	442	499	597	309	273	799	526	993	365	758	
Somali	370	335	**2036**	190	20	91	280	38	264	1208	512	23	
Tigray	24	9	29	1	0	26	282	**1940**	478	142	23	241	

The numbers highlighted in bold represent the month with the maximum disease incidence in each region

#### Meningitis disease incidence in Ethiopia from 2010 to 2022

According to Table 4, cases of meningitis were reported relatively high in December in Addis Ababa, Amhara, Benishangul-Gumuz, Gambella, and Harari. November marked the peak in Oromia and Sidama. In Afar, SNNPR, Somalia, and Tigray, the highest occurrences were recorded in June, March, July, and August, respectively.

**Fig. 7:**
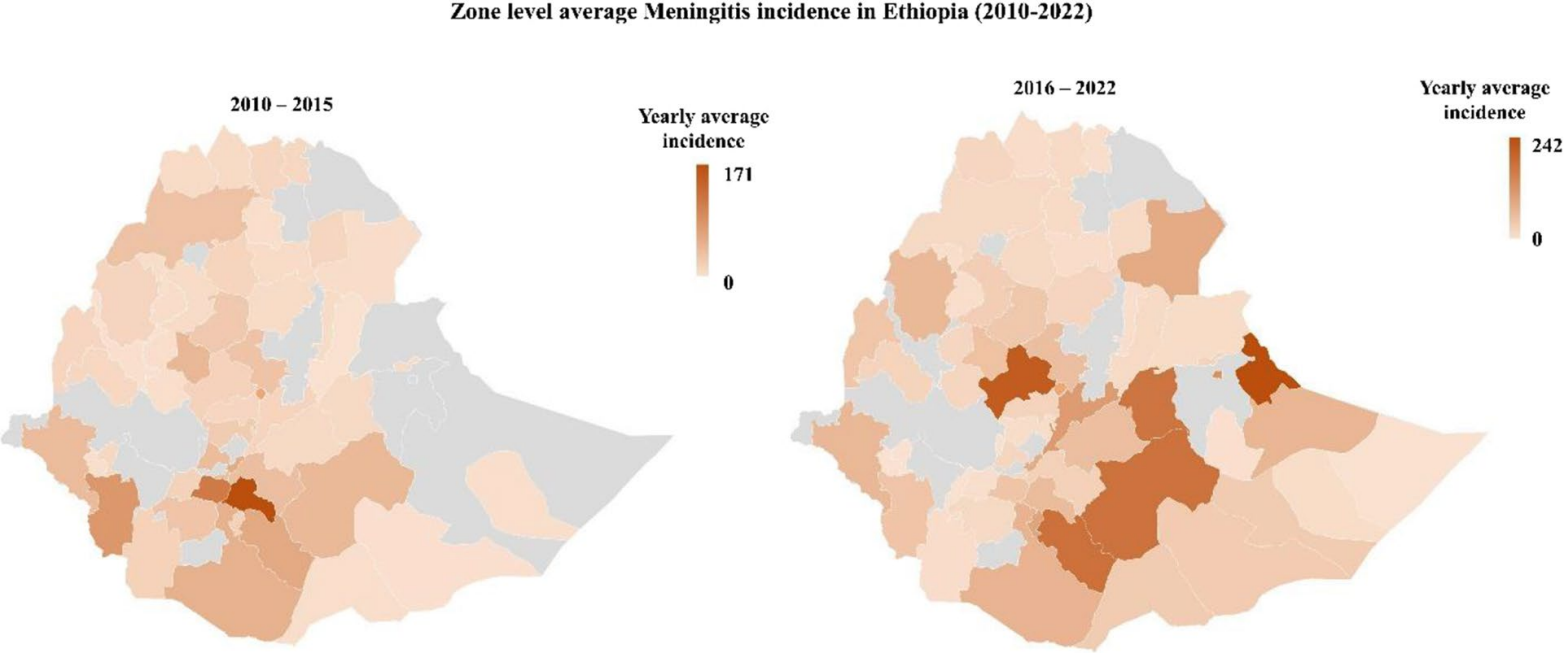
Zone level average Meningitis incidence in Ethiopia between 2010 to 2022

Figure 7 illustrates the average incidence of Meningitis at the Zone level in Ethiopia from 2010 to 2022. A more distribution has been observed in the latter period (2016–2022), in most Zones of the Somali region.

**Table 4:** Monthly regional Meningitis disease incidence in Ethiopia from 2010 to 2022

Region	Month												
	JAN	FEB	MAR	APR	MAY	JUN	JUL	AUG	SEP	OCT	NOV	DEC	
Addis Ababa	114	107	133	150	113	95	96	107	75	116	127	**167**	
Afar	68	72	75	65	69	**106**	142	67	56	61	73	62	
Amhara	224	235	281	292	275	274	179	278	261	283	366	**473**	
Benishangul-Gumuz	77	75	81	67	54	68	41	59	65	91	97	**101**	
Dire Dawa	7	1	8	3	19	20	8	22	26	30	18	24	
Gambella	74	73	56	64	69	56	49	79	57	77	73	**103**	
Harari	69	69	71	68	72	50	57	74	86	93	66	**96**	
Oromia	1025	1048	1114	1123	1187	953	1207	1150	1059	1157	**1768**	1367	
Sidama	17	29	42	33	30	34	57	41	34	59	**69**	58	
SNNPR	731	850	**1058**	925	665	653	624	701	690	744	717	726	
Somali	230	243	260	248	274	248	**365**	353	300	352	338	272	
Tigray	26	61	63	76	57	48	56	**70**	35	55	40	42	

The numbers highlighted in bold represent the month with the maximum disease incidence in each region

#### Dengue fever and leishmaniasis disease incidence in Ethiopia from 2018 to 2023

In Dire Dawa, the peak of dengue fever cases was observed in November, while in Somalia, it occurred in February, as indicated in Table 5. On the other hand, Leishmaniasis reached its highest incidence in the Amhara region in March.

**Fig. 8:**
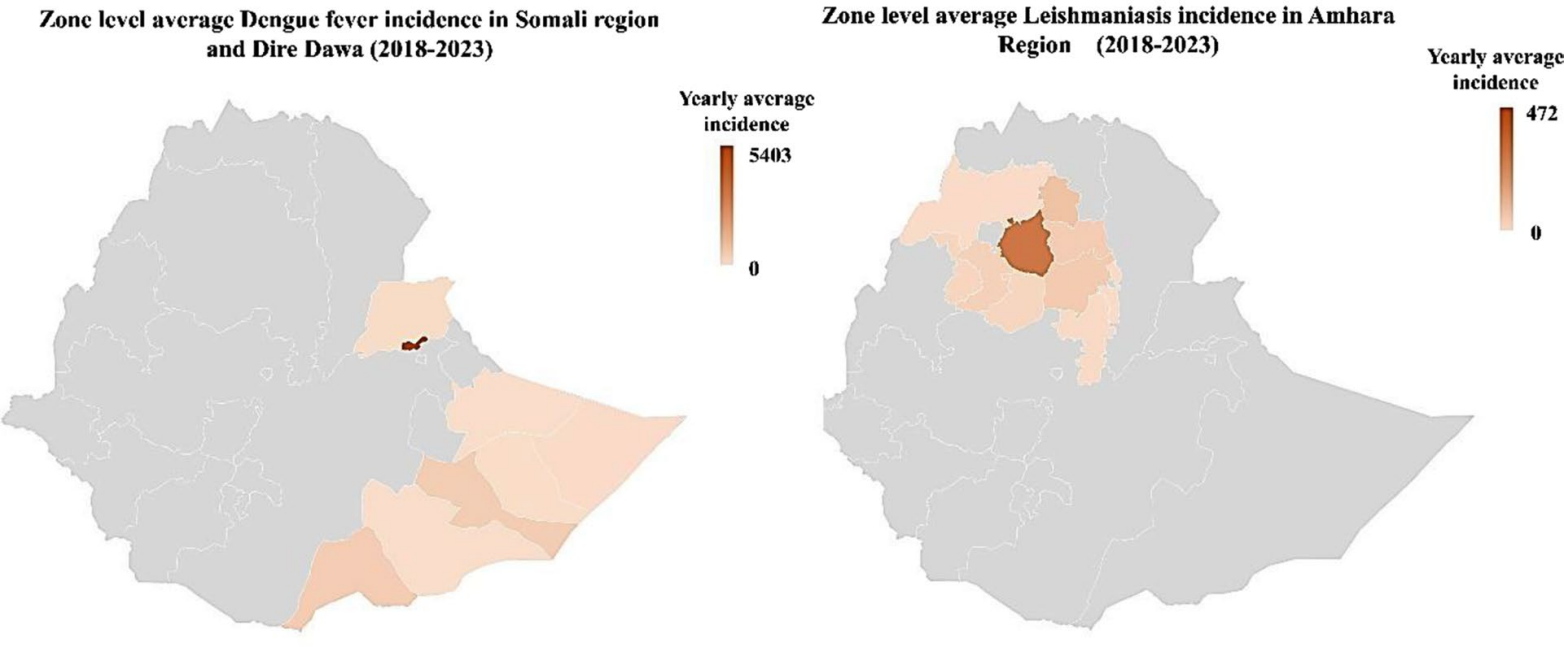
Zone level average Dengue fever and Leishmaniasis incidences in Ethiopia between 2018 to 2023

Figure 8 shows the Dengue fever and Leishmaniasis Zone level average over five years incidences in Amhara region (Leishmaniasis), Somali region and the Dire Dawa city admisntration (Dengue fever) between 2018 and 2023.

**Table 5:** Monthly Dengue Fever and Leishmaniasis disease incidence in Ethiopia from 2018 to 2023

Region	Dengue Fever											
	JAN	FEB	MAR	APR	MAY	JUN	JUL	AUG	SEP	OCT	NOV	DEC
Dire Dawa	1763	1530	1199	1312	1583	3770	2571	3039	2254	4332	**5897**	3650
Somali	647	**669**	453	596	422	457	697	506	505	560	537	341
	**Leishmaniasis**											
Amhara	1087	1278	**1507**	977	862	906	518	572	679	807	906	1029

The numbers highlighted in bold represent the month with the maximum disease incidence in each region

### Disease incidence yearly trends in Ethiopia from 2010 to 2022/2023

**Fig. 9:**
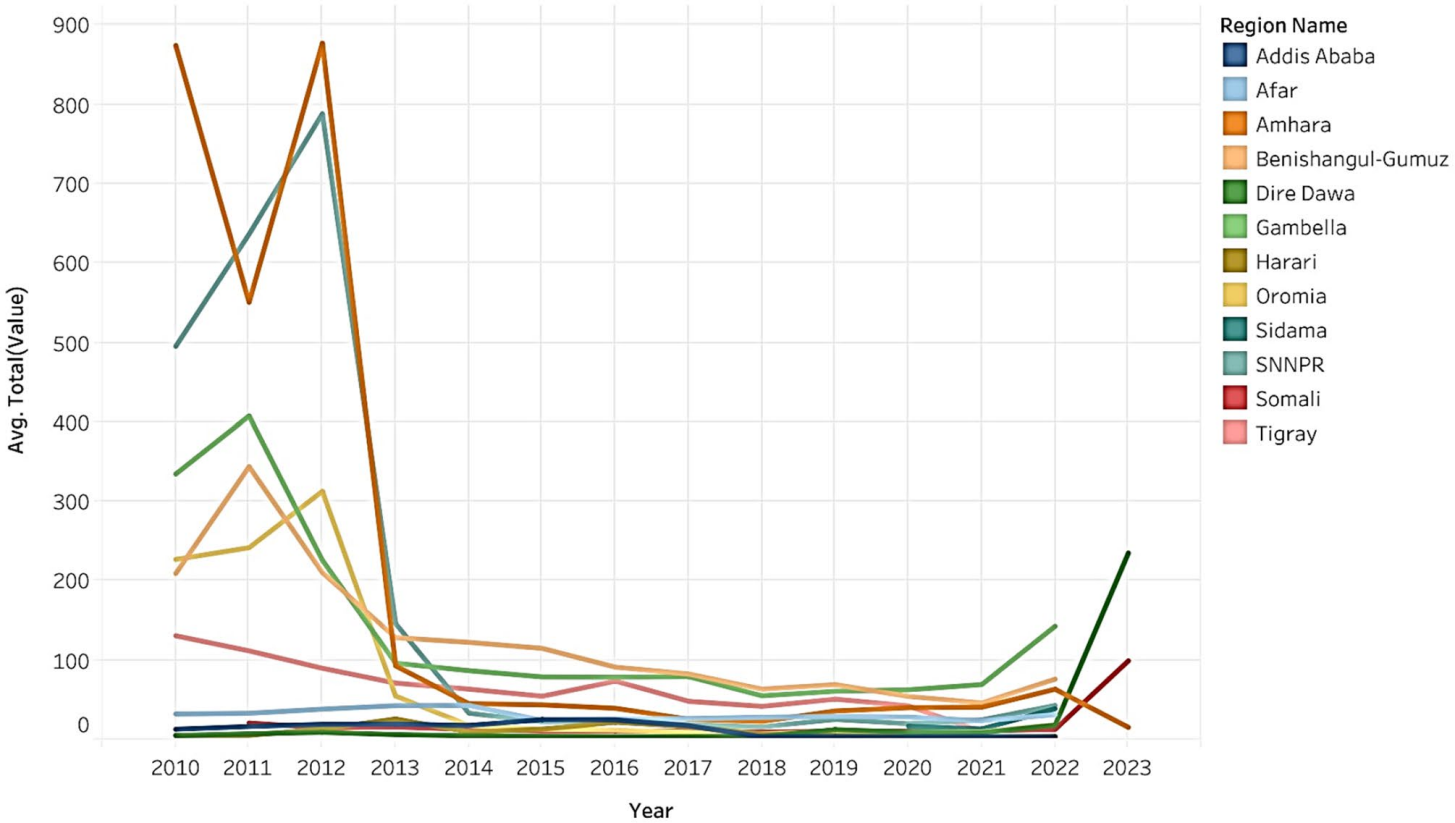
Regional yearly trends of total disease incidences in Ethiopia from 2010 to 2022/2023

**Fig. 10:**
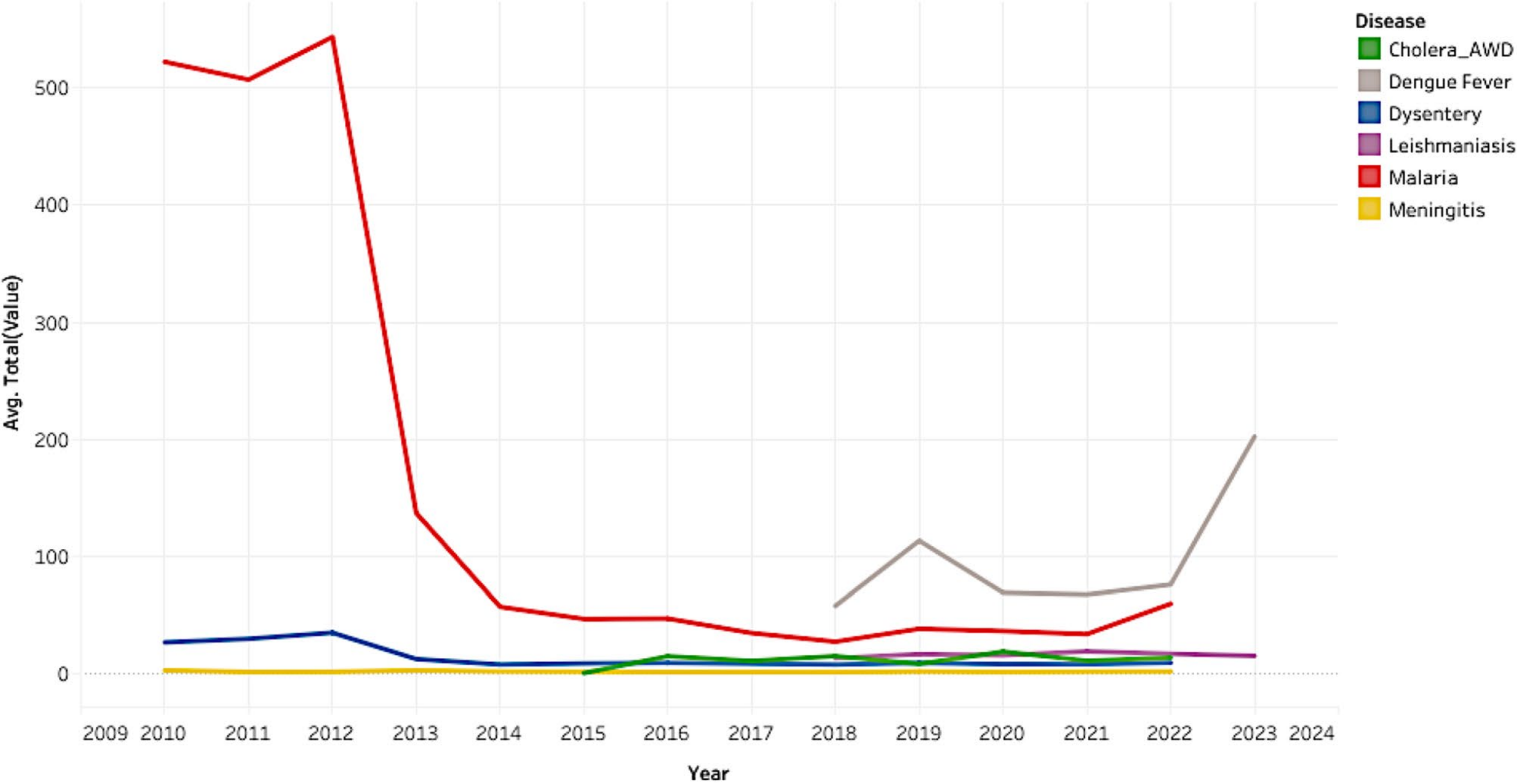
Yearly trends of total disease incidences in Ethiopia based on disease from 2010 to 2022/2023

Between the period of 2010 and 2022/2023, the highest historical disease incidence was observed in 2012 specifically in the Amhara and Afar regions for both malaria and Dysentery. Figures 9 and 10 demonstrate the regional and disease-specific yearly trends.

## Discussion

This study presents a comprehensive analysis of the spatiotemporal distribution of disease incidences in Ethiopia, encompassing six major diseases: Malaria, Meningitis, Cholera, Dysentery, Dengue Fever and Leishmaniasis. The temporal and geographic distribution of disease incidences is a critical factor in effective public health planning and intervention strategies. Through an examination of data sourced from the Ethiopian Public Health Institute’s Public Health Emergency Management (PHEM), this study provides insights into the occurrence and propagation of each disease across different regions of Ethiopia. By employing spatial mapping and time series analysis, the study identifies hotspots, trends, and seasonal variations in disease incidence.

The findings of this analysis unveil distinct patterns for each disease, characterized by varying incidences and temporal dynamics.

Malaria is influenced by a complex interplay of environmental, social, and individual factors. Environmental factors such as climate, temperature, and rainfall impact the breeding and survival of the Anopheles mosquitoes, which transmit the malaria parasite [23]. Geographical location and landscape also play a role, as areas with stagnant water bodies are more conducive to mosquito breeding (Amhara, Oromia and Sidama regions are good examples). Socioeconomic factors, including poverty and limited access to healthcare, contribute to higher malaria risk, as vulnerable populations may lack the means for preventive measures and timely treatment. Individual factors such as age, immunity, and travel history also influence susceptibility. Additionally, the prevalence of drug-resistant malaria strains poses a significant risk in some regions. Effective malaria prevention and control strategies require addressing this multifaceted range of risk factors [24]. According to our finding, a prominent cyclical pattern emerges, characterized by its peak incidence coinciding notably with the rainy and humid season. This phenomenon aligns with the well-established trend often observed in vector-borne diseases, wherein the availability of breeding sites for disease vectors is augmented by the increased humidity and precipitation during this period [25–27]. The insight gained from this cyclical pattern not only substantiates existing knowledge but also serves as a critical point of reference for timing interventions and resource allocation to effectively mitigate Malaria incidences.

Cholera/Diarrhea risk factors include poor sanitation, inadequate access to clean water, overcrowded living conditions, low socioeconomic status, and individual behaviors like poor hygiene and improper food handling. Population density and increased mobility, particularly in close and crowded settings, facilitate the rapid spread of infectious diseases, heightening the risk in certain clusters with extensive human interaction for Cholera. Unique environmental conditions, including climate and weather factors including temperature and humidity, contribute to the prevalence and transmission of these pathogens causing meningitis in specific clusters [28, 29]. Socioeconomic factors, such as poor hygiene and sanitation practices, amplify the transmission risk, particularly in clusters with inadequate sanitation infrastructure [30], which is also the case for those areas where more incidences are reported in this paper. Additionally, the presence of animal reservoirs and zoonotic transmission which can elevate the risk in clusters with proximity to animals or specific animal-human interactions, could be the reasons origins of sporadic meningitis epidemics in those areas [31, 32]. Somali, Sidama and Dire Dawa are typical examples for this. Additionally, malnutrition, weakened immune systems, and pre-existing health conditions contribute to susceptibility [33]. Limited access to healthcare facilities and delays in diagnosis and treatment exacerbate the impact of meningitis incidences, particularly in clusters with poor healthcare infrastructure, potentially leading to more severe epidemics.

Dysentery risk factors encompass poor sanitation, crowded living conditions, malnutrition, compromised immune systems, age (with children being more vulnerable), prior infections increasing susceptibility, contaminated food and water consumption, poor personal hygiene practices, and close contact with infected individuals [34]. These factors contribute to the transmission of the bacteria or parasites responsible for dysentery, emphasizing the importance of preventive measures such as maintaining good hygiene, ensuring access to clean water, and avoiding contaminated food sources. Dysentery showcases a persistent presence throughout the study years. Given the consistent occurrence of Dysentery cases, it is evident that effective strategies to combat this ailment necessitate not only reactive responses but also a proactive approach centered around preventive measures, health education, and hygiene awareness campaigns.

When considering the geographical distribution, it becomes evident that the regions of Amhara, Sidama, SNNPR and Oromia are characterized by their substantial population densities, exhibit a pronounced prevalence of disease incidences during the period spanning from 2010 to 2022/2023. This heightened incidence within these regions can be attributed to their large and densely populated communities, which potentially create an environment conducive to disease transmission and propagation. The convergence of sizable populations with various social and environmental factors likely contributes to the increased vulnerability and susceptibility to disease incidences in these areas [35, 36]. Thus, the observation of heightened disease incidence in Amhara, Sidama SNNPR and Oromia underscores the intricate interplay between demographic dynamics and disease prevalence, shedding light on the necessity for tailored public health interventions and resource allocation. On the other hand, dengue fever exhibits a predominant prevalence within the eastern parts of Ethiopia, characterized by their hot climates and extended periods of drought. This observation aligns with the established understanding that dengue fever thrives in warm, tropical environments, giving rise to flu-like symptoms and, in some instances, progressing to a severe manifestation known as severe dengue [37].

Efforts to address these climate-sensitive diseases particularly in Ethiopia and Sub-Saharan countries, in general, require a multi-faceted approach. This includes strengthening healthcare systems, improving sanitation and water management, implementing effective vector control measures, enhancing disease surveillance, and promoting climate adaptation strategies to mitigate the impact of changing environmental conditions on public health.

In summary, this study serves as a foundational resource for evidence-based decision-making in disease prevention and control strategies. The complete investigation of the spatiotemporal dynamics of disease incidences yielded a detailed understanding of disease distribution that equips health authorities to engage pro-actively in preserving public health in the Ethiopian environment. By providing evidence-based insights into the spatio-temporal patterns associated with each disease, this study provides health authorities with the necessary tools to make informed decisions in the prevention and control of diseases. The findings of this study have the potential to guide policymaking and resource allocation, facilitating proactive measures that mitigate the repercussions of incidences and ensure the well-being of the population. The incorporation of these findings into public health policy can open the door for efficient interventions and, in the end, help to lessen the effects of disease on communities throughout the region.

## Conclusion

Exploratory analysis illuminates the spatio-temporal dynamics of disease incidence distribution in Ethiopia, encompassing six significant diseases over a 13-year span. The distinct patterns observed for each disease, coupled with their varying prevalence and temporal behaviors, underscore the need for tailored interventions. The recurring pattern of Malaria underscores the significance of prompt interventions, whereas the continual occurrences of Dysentery underline the need for vigilance. The enduring cases of dengue fever in eastern Ethiopia and instances of Leishmaniasis in the Amhara region emphasize the ongoing importance of preventive measures and underscore the necessity for preparedness. The evident correlation between disease prevalence and regions with substantial populations, such as Oromia and Amhara, signifies the necessity for tailored interventions within these densely inhabited areas. The identification of high-risk regions provides a strategic foundation for resource allocation, targeted interventions, and proactive measures to mitigate outbreaks’ impact. By integrating these insights into policy and practice, Ethiopia can enhance its public health approach, effectively mitigating disease impact and fostering a more resilient society.

## Data Availability

The datasets used and/or analyzed during the current study are available from the corresponding author up on a reasonable request and with a written permission from the original data sources.
